# Image-Derived Blood Normalization of Antibody-Based TREM2 PET in Mouse Models of Amyloidosis and Myocardial Infarction

**DOI:** 10.2967/jnumed.125.269472

**Published:** 2025-09

**Authors:** Rebecca Schaefer, Lea H. Kunze, Marlies Haertel, Yeqian Zhu, Kai Schlepckow, Michael Willem, Laura M. Bartos, Giovanna Palumbo, Lukas Tomas, Christian Schulz, Steffen Massberg, Rudolf A. Werner, Maximilian Fischer, Dan Xia, Kathryn M. Monroe, Christian Haass, Matthias Brendel, Simon Lindner

**Affiliations:** 1 Department of Nuclear Medicine, LMU University Hospital, LMU Munich, Munich, Germany;; 2 Institute of Surgical Research at the Walter-Brendel-Centre of Experimental Medicine, University Hospital, LMU Munich, Munich, Germany;; 3 German Center for Neurodegenerative Diseases (DZNE), Munich, Germany;; 4 Chair of Metabolic Biochemistry, Biomedical Center (BMC), Faculty of Medicine, LMU Munich, Munich, Germany;; 5 Department of Medicine I, LMU University Hospital, LMU Munich, Munich, Germany;; 6 German Centre for Cardiovascular Research (DZHK), Partner Site Munich Heart Alliance, Munich, Germany;; 7 Department of Immunopharmacology, Mannheim Institute for Innate Immunoscience (MI3), Medical Faculty Mannheim, Heidelberg University, Mannheim, Germany;; 8 Russell H. Morgan Department of Radiology and Radiological Sciences, Johns Hopkins School of Medicine, Baltimore, Maryland;; 9 Denali Therapeutics Inc., South San Francisco, California;; 10 Munich Cluster of Systems Neurology (SyNergy), Munich, Germany; and; 11 German Cancer Consortium (DKTK), Partner Site Munich, German Cancer Research Center, Heidelberg, Germany

**Keywords:** TREM2, PET, ATV:4D9, ^64^Cu, SPM

## Abstract

The triggering receptor expressed on myeloid cells 2 (TREM2) plays a pivotal role in the activation of myeloid cells and is currently being investigated as a potential therapeutic target in several diseases. In this study, we established enhanced quantification of PET images of a ^64^Cu-labeled antibody-based PET radiotracer as a noninvasive tool for the assessment of TREM2 expression in the brain and peripheral organs of mice. We used TREM2 knockout mice that lack target expression to investigate data-driven blood normalization of PET images against percentage of injected dose normalization. **Methods:** TREM2 knockout and wild-type mice (*n* = 11 each) were injected with the radiotracer [^64^Cu]Cu-NODAGA-ATV:4D9 (ATV is antibody transport vehicle). Twenty hours after injection, TREM2 PET was conducted and blood samples were collected. A voxelwise analysis with statistical parametric mapping served to determine voxels that correlate with ex vivo blood radioactivity levels. Furthermore, TREM2 PET signals were compared between mice with and those without TREM2 expression using image-derived blood normalization. Correlation with TREM2 protein expression levels in the lung, liver, spleen, and bone marrow was used to validate organ-specific PET results. Disease models of brain amyloidosis and myocardial infarction were investigated to test for the value of image-derived normalization in mice. **Results:** Blood radioactivity levels derived from a statistical parametric mapping–derived region of interest demonstrated a robust correlation with radioactivity measurements obtained from ex vivo blood samples. Voxelwise clusters of TREM2 PET signals were more robustly detected after blood normalization of the PET images. Significant voxelwise clusters of TREM2 PET signals in peripheral organs correlated with TREM2 protein expression levels. Furthermore, image-derived normalization enhanced the significance of voxelwise clusters of TREM2 in the brains of *App*^SAA^;TfR^mu/hu^ mice, as well as the TREM2 signal in the myocardial infarct region. Both strongly correlated with ex vivo autoradiography. **Conclusion:** Normalization of PET images to account for blood levels enhanced the detection of TREM2. This improved methodology for TREM2 PET analysis provides a promising basis for future assessments of TREM2 imaging.

## Visual Abstract

**Figure:**
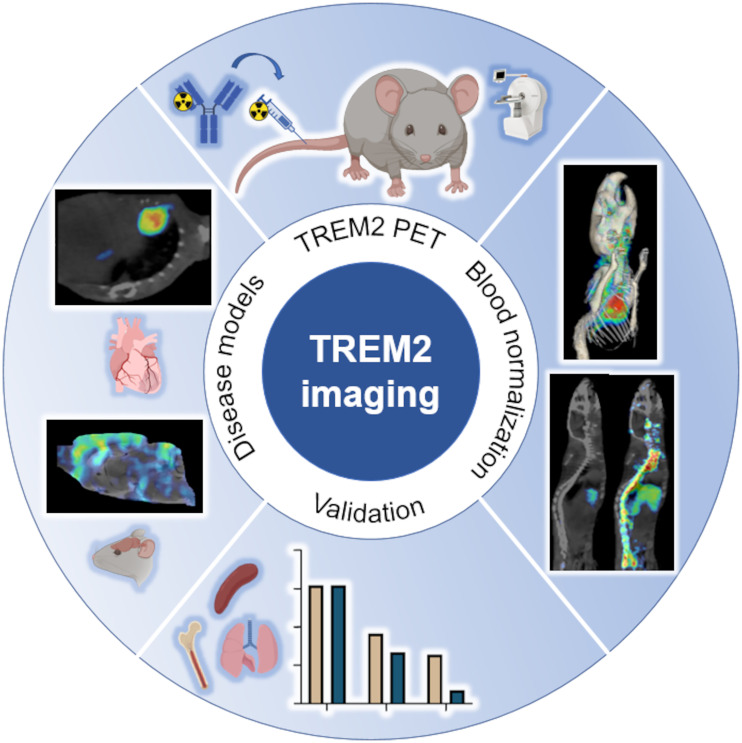

Alzheimer disease (AD) and other neurodegenerative disorders represent significant public health challenges, particularly in an aging society, and require development of novel diagnostic and therapeutic approaches. In addition to accumulation of β-amyloid peptide and fibrillary tau aggregates, AD is also associated with microgliosis. Microglia are resident macrophages of the central nervous system (CNS). They exist in a range of cellular states from homeostatic to diverse responsive states (1,2). Triggering receptor expressed on myeloid cells 2 (TREM2) is a key player in the regulation of microglial responses to pathologic challenges and is selectively expressed in microglia in the CNS (3).

Furthermore, TREM2 is expressed in the periphery of myeloid-derived dendritic cells and tissue macrophages, including osteoclasts in the bone, as well as in subsets in the liver, adipose tissue, skin, gut, and tumors (4), which suggests its involvement in diverse physiologic and pathologic processes beyond the CNS. Emerging evidence indicates that TREM2 may be involved in peripheral inflammation (5), bone metabolism (6), and lipid homeostasis (7), thus expanding its relevance to a broader range of diseases, including metabolic disorders and inflammatory conditions. PET constitutes a valuable tool to noninvasively visualize and quantify TREM2 expression in vivo with high sensitivity throughout the body.

Recently, we developed a radiotracer using a ^64^Cu-labeled antibody, [^64^Cu]Cu-NODAGA-ATV:4D9 (ATV is antibody transport vehicle), which uses the human transferrin receptor as a blood–brain barrier transport vehicle for a TREM2 antibody (8) to image TREM2-associated microglial activation in the CNS of an AD mouse model using PET (9).

Although PET imaging has demonstrated promising results for the detection of TREM2-expressing microglia, optimizing the quantification methods remains a critical step for accurate assessment and translation toward human imaging. Typically, PET images are normalized to the percentage of injected dose (%ID) of the radiotracer; however, this approach may not fully account for individual variations in the tracer pharmacokinetics (i.e., clearance from the organism). In this study, we aimed to improve the quantification of TREM2 PET signals by exploiting mice with TREM2 knockout as proper controls. We modified the protocol for PET image analysis, using blood normalization instead of %ID normalization, as previously described (9). We hypothesized that normalizing PET images to blood levels rather than to %ID would yield improved results for the detection and quantification of TREM2 expression. Normalizing PET images to the blood activity concentration rather than the injected dose has the advantage of accounting for physiologic variations in metabolism, blood volume, and organ function. This approach is especially beneficial when the drug is unevenly distributed because of differences in organ function or pathologic conditions. This method provides a more accurate representation of the target binding of the radiotracer because it subtracts radioactivity levels in the blood. We correlated TREM2 PET results with gold standard TREM2 protein expression levels in the lung, liver, spleen, and bone marrow and compared TREM2 PET images of the brain and heart with results obtained by ex vivo autoradiography in mouse models of AD and myocardial infarction, respectively.

## MATERIALS AND METHODS

The full version of the materials and methods section is provided in the supplemental materials (available at http://jnm.snmjournals.org).

### Animals

All experiments were conducted in accordance with the animal protection laws of the state of Upper Bavaria (Germany) and with the approval of the regional animal care committee (Regierung von Oberbayern, ROB-55.2-2532.Vet_02-21-156, ROB-55.2-2532.Vet_02-19-26, ROB-55.2-2532.Vet_02-19-17), supervised by a veterinarian.

The following cohorts of mice were selected: (cohort 1A) C57BL/6J, referred to as wild-type (WT) mice (*n* = 11); (cohort 1B) TREM2 knockout mice (*n* = 11) (10); (cohort 2A) amyloid precursor protein with SAA mutation (*App*^SAA^) mouse model; transferrin receptor (TfR^mu/hu^) (*n* = 6); (cohort 2B) WT;TfR^mu/hu^ (*n* = 6); (cohort 3) WT mice (*n* = 10).

*App*^SAA^ mice display age-dependent accumulation of amyloid plaques and elevated levels of TREM2 (11). Mice from cohorts 2A and 2B expressed the human transferrin receptor.

### Antibody

ATV:4D9 (8) antibody materials were provided by Denali Therapeutics. ATV:4D9 is a bispecific antibody with murine anti-TREM2 clone 4D9 Fab fragments (12) and engineered Fc domain that binds the human transferrin receptor.

Since the ATV moiety is not murine cross-reactive (13) and targets only the human TfR, the ATV technology was only effective in the mice of cohorts 2A and 2B in this study.

### Radiochemistry

ATV:4D9 was modified and radiolabeled as previously reported (9). Briefly, ATV:4D9 was mixed with [^64^Cu]CuCl_2_ in ammonium acetate buffer. The radiochemical yield (*n* = 7) was 76% ± 6%, with a radiochemical purity of greater than 99% and a molar activity of 176 ± 8 MBq/nmol at the end of the synthesis.

### Small-Animal PET

[^64^Cu]Cu-NODAGA-ATV:4D9 was administered in mice via intravenous tail injection with activities of 34 ± 6 MBq for cohort 1, 15 ± 1 MBq for cohort 2, and 29 ± 1 MBq for cohort 3. Twenty hours after injection, mice underwent a PET/CT scan. CT and PET images were aligned and coregistered. Average images were generated. A sphere was manually positioned over the heart as a volume of interest (VOI) to segment blood radioactivity levels. A data-driven cortical-cluster VOI derived from statistical parametric mapping (SPM) analysis was used for segmentation of tracer uptake. In the PET images of myocardial infarcts, blood levels were manually segmented from the carotid artery, and the infarct region with the strongest signal was manually defined as a region of interest. Activity and area were extracted.

### Blood Sampling and Activity Measurements

Blood samples were collected intracardially, and activity and mass measurements were conducted using a gamma counter. Radioactivity in the blood was density corrected using the factor 1.057 g/cm^3^ (14). Results are reported as becquerels per gram and %ID per gram.

### Ex Vivo Autoradiography

*App*^SAA^;TfR^mu/hu^ mice were perfused with paraformaldehyde, and WT mice from the myocardial infarct cohort were perfused with phosphate-buffered saline. Brains were isolated, fixed, and cut into sagittal slices. Hearts were excised and sectioned axially. Sections were exposed to a phosphor imaging plate.

### Immunohistochemistry

Sections of *App*^SAA^;TfR^mu/hu^ mice were stained for β-amyloid (NAB228), Iba1, and TREM2. Quantification was conducted in 5 plaque-associated regions in the cortex and basal ganglia per mouse, measuring the TREM2-positive area within Iba1-positive microglia.

### Quantification of TREM2 Protein Levels

TREM2 levels were quantified by a Meso Scale Discovery (MSD) immunoassay as previously described (8). In brief, streptavidin plates were blocked and coated with biotinylated anti-TREM2 antibody. Lung, liver, and spleen were collected after phosphate-buffered saline perfusion from WT mice. Tissue samples and standard were diluted and added to the plates. Primary antibody was added. Plates were washed, and the primary detection antibody was added. Plates were developed, and the resulting signal was detected and converted to TREM2 concentrations. Bone marrow TREM2 protein levels were measured in another batch of animals.

### SPM

Voxelwise SPM analysis (Wellcome Department of Cognitive Neurology) was conducted using SPM12 in MATLAB version 2011-R2016 (MathWorks) (15). A voxelwise comparison analysis was performed on images with radiotracer blood levels derived from biodistribution as a vector. A data-driven cluster VOI was derived from the SPM analysis and used for the segmentation of radioactivity blood levels from PET images. PET images, normalized to SPM-derived tracer blood levels or %ID, were subjected to a voxelwise group comparison. *T* values of the lung, liver, spleen, and bone marrow were segmented using Imalytics Preclinical software (Gremse-IT GmbH) (16). To avoid spill-over signals from the surrounding bone, a sphere was positioned over the lung. For the bone marrow, a region was selected in the upper thoracic vertebrae.

### Left Anterior Descending Artery Ligation

Myocardial infarction was induced by surgical ligation of the left anterior descending artery, as described previously (17). Thoracotomy was performed, and the left anterior descending artery was ligated. Sham mice underwent the same treatment, except for the left anterior descending artery ligation.

### Statistical Analysis

All statistical analyses were performed using GraphPad Prism version 10. Data-driven analysis was performed using SPM with SPM12 routines implemented in MATLAB (version 2016) (15). Data-driven clusters were selected at a threshold of *P* that was less than 0.005 uncorrected and a cluster size of more than 50 voxels. Groups were analyzed using unpaired *t* tests (2-tailed) or correlation analyses. Statistical significance was considered at *P* values of less than 0.05 (*), less than 0.01 (**), less than 0.001 (***), and less than 0.0001 (****).

## RESULTS

### Correlation Between Blood Radioactivity Ex Vivo and PET Radioactivity

**FIGURE 1.**
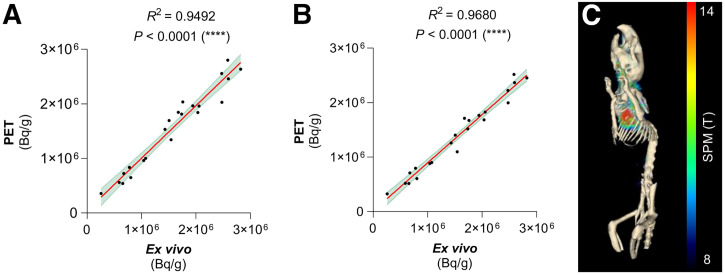
Correlation between radioactivity concentration in ex vivo blood samples and radioactivity concentration derived from manually placed VOIs (A) or SPM-driven VOIs (B) in PET images of WT and TREM2 knockout mice (*n* = 11 each). Linear regression, 95% CI. Signals resolved for genotype and correlations are presented in Supplemental Figure 4. (C) SPM image correlating individual PET images and ex vivo blood levels projected on CT. Multiple regression. Resulting SPM image with *P* < 0.05 and cluster size of >50 voxels is presented in Supplemental Figure 2B.

A significant positive correlation was observed between radioactivity measurements of ex vivo blood samples and blood level concentrations derived from PET from a manual VOI placed over the heart (correlation coefficient [*R*^2^] = 0.949; *P* < 0.0001; slope, 0.963) (Fig. 1A) or an SPM-derived VOI over the heart (*R*^2^ = 0.968; *P* < 0.0001; slope, 0.884) (Fig. 1B). To assess potential biases, a Bland–Altman analysis was performed, revealing a bias of 17,423 Bq/g (SD, 171,008 Bq/g) for manually segmented PET data and a higher bias of 171,406 Bq/g (SD, 150,279 Bq/g) for SPM-driven segmentation, suggesting a more systematic offset but lower variability (Supplemental Fig. 1). The 3-dimensional image of voxels in the PET images that correlate with ex vivo blood radioactivity levels (Fig. 1C) provides a visual representation of the vascular tree. The image-derived VOIs for the radioactivity levels in the blood derived from SPM analysis in the heart, and an alternative VOI in the carotid artery, are presented in Supplemental Figure 2.

Radioactivity concentrations derived from PET showed no correlation with age, sex, or weight (Supplemental Fig. 3).

### Whole-Body Voxelwise Comparison Between Mice With and Without TREM2

**FIGURE 2.**
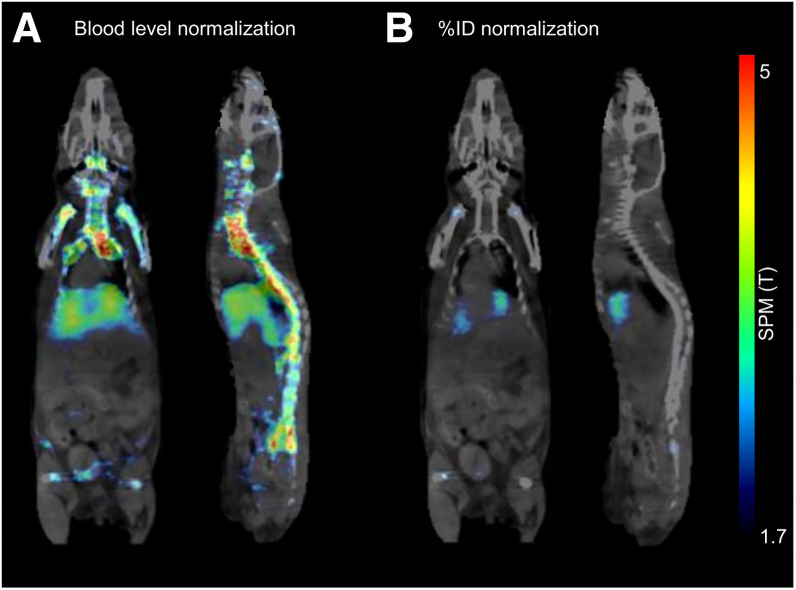
Voxelwise group comparison of TREM2 radiotracer uptake in PET images of WT vs. TREM2 knockout mice (*n* = 11 each) after SPM-derived blood level normalization (A) or %ID normalization (B). Two-sample *t* test, *P* < 0.05, cluster size of >50 voxels projected onto CT. Average PET images are presented in Supplemental Figure 5.

The results of the SPM analysis of the group comparison between WT and TREM2 knockout mice represent the spatial differences in radioactivity signal between the 2 groups. Voxelwise group contrasts between WT and TREM2 knockout mice revealed an enhanced signal difference for image-derived blood normalization in comparison to %ID (Fig. 2). Liver and bone exhibited the strongest differences of TREM2 tracer uptake between WT and TREM2 knockout mice (Fig. 2).

### Correlation of TREM2 Levels Between SPM and Protein Expression

**FIGURE 3.**
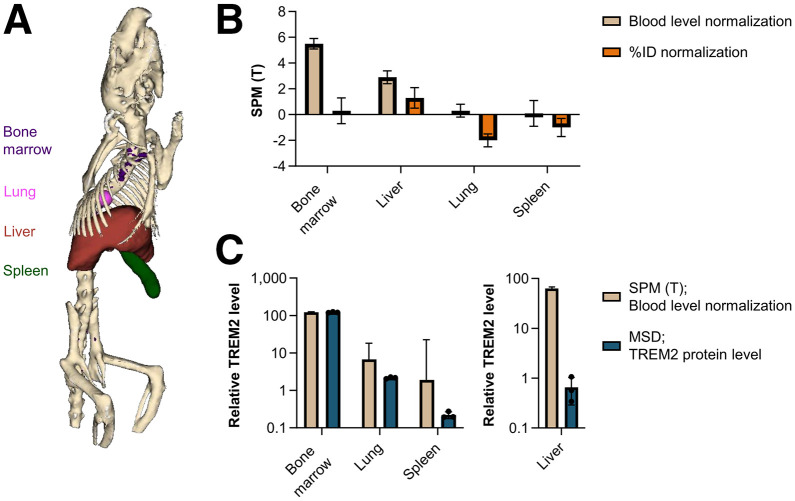
(A) 3-dimensional CT-based segmentation of mouse, displaying bones (beige), bone marrow (purple), lung (pink), liver (brown), and spleen (green). (B) *T* values derived from SPM images of group comparisons of WT vs. TREM2 knockout mice (*n* = 11 each) using SPM-derived blood normalization or %ID normalization. Mean ± SD. (C) *T* values derived from blood normalization from (B) and mouse TREM2 protein levels detected by MSD in WT mice (*n* = 3). Mean ± SD.

Group difference *T* values for bone marrow, liver, lung, and spleen were derived from SPM analysis and exhibited higher values when PET images were normalized to blood levels in comparison to %ID normalization (Fig. 3; Supplemental Tables 1 and 2). The lung and spleen yielded negative *T* values from SPM for %ID-normalized PET images. The *T* values from SPM analysis of blood-level–normalized PET images for bone marrow, lung, and spleen exhibited relative alignment with TREM2 levels determined by MSD analysis (Fig. 3C; Supplemental Table 3) and significant correlation (Supplemental Fig. 6). The *T* value of the liver was considerably higher than the MSD-derived value for TREM2 level. Correlation between the difference in PET of WT versus TREM2 knockout mice after blood-level normalization and TREM2 protein levels from MSD analysis (excluding the liver values) revealed a strong correlation (*R*^2^ = 0.9950; Supplemental Fig. 7).

### Voxelwise Brain Analysis of Amyloid Mice

**FIGURE 4.**
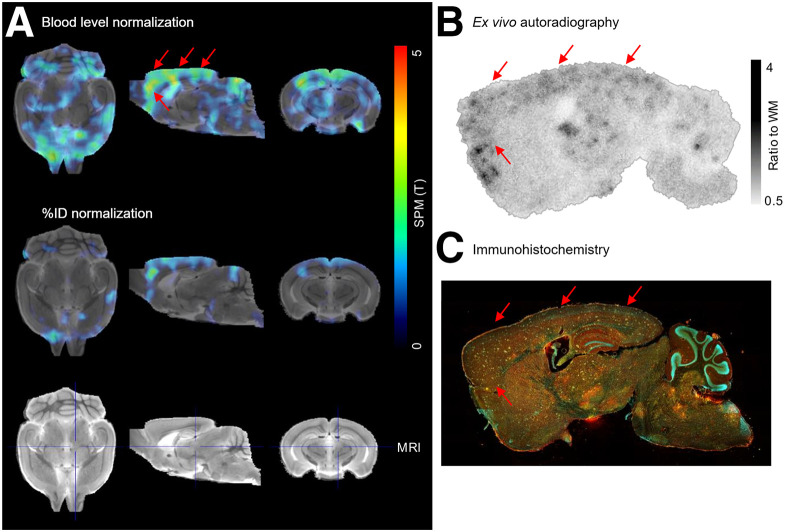
(A) Voxelwise group comparison of TREM2 radiotracer uptake in brains of *App*^SAA^;TfR^mu/hu^ vs. WT;TfR^mu/hu^ mice (*n* = 6 each) of PET images after SPM-derived blood level normalization or %ID normalization. Two-sample *t* test, *P* < 0.05, cluster size of >50 voxels, projected onto a mouse MRI. Average PET images are presented in Supplemental Figure 11. (B) Ex vivo autoradiography of representative *App*^SAA^;TfR^mu/hu^ brain slice. Additional images are available in Supplemental Figure 12. (C) Immunohistochemistry of adjacent brain slice (DNA/4′,6-diamidino-2-phenylindole [blue], microglia/Iba1 [yellow], and TREM2 [red]). Individual fluorescence channels are shown at plaque level in Supplemental Figure 13. Red arrows indicate TREM2-rich signals. WM = white matter.

Voxelwise group contrasts of the brains between *App*^SAA^;TfR^mu/hu^ and WT;TfR^mu/hu^ mice demonstrated an enhanced signal difference for blood normalization compared with %ID (Fig. 4A). The strongest difference in TREM2 radiotracer uptake was observed in the cortex and in the hippocampus. Cortical TREM2 radiotracer uptake in ex vivo autoradiography of *App*^SAA^;TfR^mu/hu^ mice strongly correlated with the signal of voxelwise group contrasts of SPM regions (Fig. 4B), resulting in cortex–to–white matter ratios of 1.9 (Supplemental Fig. 8). TREM2 radiotracer uptake in the cortex was 1.4-fold higher in PET images of *App*^SAA^;TfR^mu/hu^ mice compared with that of WT;TfR^mu/hu^ mice after normalization to %ID (*P* = 0.0123) and 1.7-fold higher after normalization to blood (*P* = 0.0045) (Supplemental Fig. 9). Further regional quantification of TREM2 expression revealed significantly higher TREM2 radiotracer uptake in the cortex compared with the basal ganglia in ex vivo autoradiography (2.0-fold) and immunohistochemistry (2.6-fold) (Supplemental Figs. 10A and 10B). Immunohistochemistry-derived TREM2 expression strongly correlated with the ex vivo autoradiography signal (*R*^2^ = 0.6902) (Supplemental Fig. 10C) and colocalized with the signal of voxelwise group contrast of SPM regions (Fig. 4C).

### TREM2 Imaging of Myocardial Infarction in Mice

**FIGURE 5.**
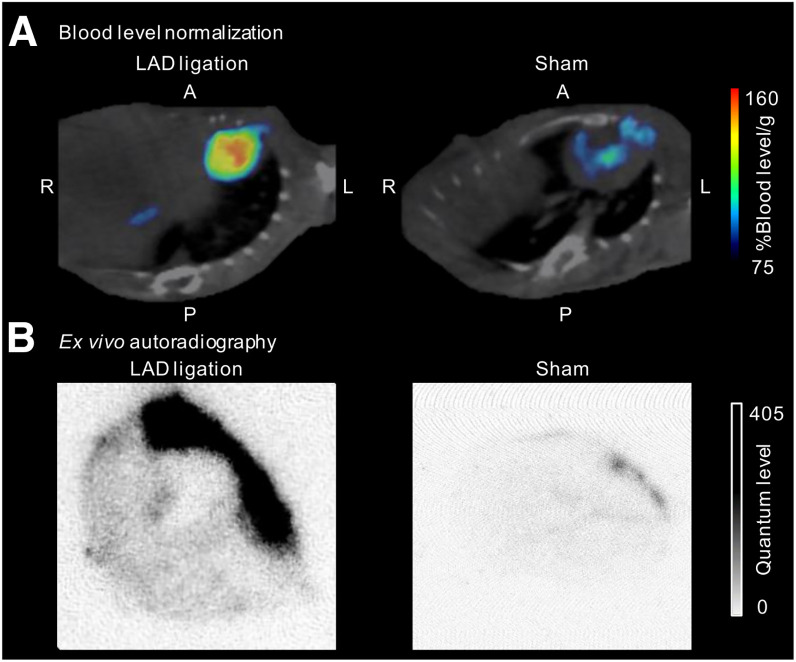
(A) Representative PET images of mice with left anterior descending (LAD) ligation and sham mouse, normalized to blood levels using segmentation from carotid artery. (B) Corresponding ex vivo autoradiography images. Quantification of tracer signal in PET and ex vivo autoradiography is available in Supplemental Figure 15. A = anterior; P = posterior.

The correlation between radioactivity levels in the infarct region and the corresponding ex vivo autoradiography was more significant when the PET images were normalized to blood levels (*R*^2^ = 0.858) compared with normalization by %ID (*R*^2^ = 0.805) (Supplemental Fig. 14). Segmented blood levels were derived from the carotid artery to circumvent spill-in signals from the infarct wall. PET images demonstrated minimal signal intensity in sham mice, whereas mice with myocardial infarction exhibited a markedly elevated signal (Fig. 5A). Notably, 2 mice exhibited markedly higher TREM2 tracer signals, which coincided with significantly enlarged hearts (approximately 1.5-fold increase in size). Furthermore, the TREM2 PET patterns in myocardial infarction colocalized with the ex vivo autoradiography signals, whereas sham mice exhibited only a minimal signal (Fig. 5B).

## DISCUSSION

TREM2 has become a significant molecular biomarker, and its potential applications as a diagnostic tool, prognostic indicator, and potential therapeutic target are being elucidated across various conditions (18) because of its regulation of key macrophage and microglia functions in disease. Following the modification of standardized PET image analysis protocols to circumvent signals in PET images from the blood levels of a radiotracer with slow kinetics, we observed robust TREM2 signals in the brains and hearts of a mouse model of myocardial infarction and peripheral organs of mice. The results of the PET analysis were validated by ex vivo autoradiography and compared with TREM2 protein expression in the lung, liver, spleen, and bone marrow. These findings provide a basis for PET monitoring of therapeutic agents that target TREM2 and microglial activation in the CNS and TREM2 expression in the periphery of mice.

The blood levels of radiotracer obtained from the segmentation of PET images through data-driven analysis using SPM exhibited a stronger correlation and lower variability in difference with radioactivity measurements derived from ex vivo blood sampling than those derived through manual PET image segmentation (Fig. 1; Supplemental Fig. 1). However, the segmentation of radiotracer levels in the blood slightly underestimated blood levels, which is likely attributable to imperfect spatial alignment of the heart due to the varying body size of the mice. Additional sources of bias may include partial-volume effects and calibration discrepancies between the methods. Although both methods produce similar results, data-driven segmentation offers significant advantages in terms of speed, reproducibility, scalability, and robustness, making it a reliable alternative to ex vivo blood sampling. TREM2 knockout mice exhibited blood levels higher than those of WT mice, likely because of the lack of target binding.

Voxelwise group comparisons of the traditional %ID normalization versus the new blood-normalization method of whole-body PET scans demonstrated a more pronounced radioactivity signal difference for the bone and liver in the blood-normalized method (Fig. 2), which is consistent with the expression of TREM2 in those organs.

The *T* values derived from SPM analysis of blood-normalized PET images confirmed the observation of a robust signal for bone marrow and the liver, as well as for the lung and spleen, in WT mice in comparison to TREM2 knockout mice (Fig. 3). The *T* values of the %ID-normalized PET images exhibited negative values for the lung and spleen, indicating a higher signal in these regions in TREM2 knockout mice compared with WT mice. The *T* values after blood normalization were also close to 0 (Supplemental Table 1), and the negative *T* values after %ID normalization (Supplemental Table 2) may be attributed to the limitation of PET resolution. The *T* values of blood-normalized PET images exhibited a strong correlation with TREM2 protein expression in the bone marrow, lung, and spleen (Fig. 3), as well as the differences in WT versus TREM2 knockout PET after blood normalization (Supplemental Fig. 7). However, this correlation is largely driven by the high TREM2 expression in the bone marrow. The considerably higher *T* value observed for the liver may be a result of the liver signal in PET being predominantly due to excretion rather than TREM2 binding. This phenomenon is also evident in the group average images of PET (Supplemental Fig. 5). TREM2 knockout mice as controls may overestimate the correlation effects because of the complete absence of TREM2. However, literature data indicate an approximately 5-fold increase in TREM2 levels in the *App*^SAA^;TfR^mu/hu^ (11) and myocardial infarction models (19). The relative increase is considerably smaller than the relative increase in the case of complete absence of TREM2 in TREM2 knockout mice. This supports that differences in results between WT and TREM2 knockout mice are not solely driven by extreme group separation, supporting the validity of our findings.

Voxelwise group comparisons of the brain PET images of *App*^SAA^;TfR^mu/hu^ and WT;TfR^mu/hu^ mice, conducted using %ID normalization versus blood normalization, demonstrated a more pronounced radioactivity signal difference in the cortex and hippocampus (Fig. 4), which colocalized with ex vivo autoradiography and immunohistochemistry and was consistent with the neuropathology observed in *App*^SAA^ mice. Amyloid plaques are observed in *App*^SAA^ homozygous mice at 4 mo of age. These plaques are present in multiple brain regions, with the most notable neuropathology occurring in the cortex and hippocampus and accompanied by microgliosis (11).

Postmyocardial infarction PET images revealed an increase in radioactivity signals within the infarct area, which correlated with the corresponding ex vivo autoradiography images (Fig. 5). Variability in tracer signal within the infarct region is likely due to variability in surgical ligation placement, leading to more extensive ischemic damage and inflammation (20). The elevated signal was localized in the infarct zone, suggesting a stronger macrophage-driven immune response driven by a larger infarct size, increased inflammation, or maladaptive remodeling. Activation of TREM2 has been demonstrated to facilitate the phagocytosis of apoptotic cells and debris, thereby contributing to the clearance of necrotic tissue in a range of tissues, including the brain (21–23). It is plausible that a similar mechanism may take place in the context of myocardial infarction. It has recently been shown that TREM2 expression is significantly upregulated in cardiac tissue after myocardial infarction in mice (19,24). The imaging timeline was designed to capture peak TREM2 expression, which occurs around day 5 after myocardial infarction (25), with sustained expression beyond this phase (19), allowing ex vivo validation on day 7 while still reflecting the relevant TREM2 levels. These findings support the myocardial infarction model as a pilot for studying peripheral TREM2-related disease processes and its role in postmyocardial infarction immune responses.

Blood activity levels did not show significant differences between *App*^SAA^;TfR^mu/hu^ and WT;TfR^mu/hu^ mice, myocardial infarction and sham mice, or WT and TREM2 knockout mice, suggesting that pathology-driven changes in circulating tracer levels were minimal in our models (Supplemental Fig. 16).

Some limitations have to be considered for this study. The radiotracer targets both membrane-bound TREM2 and soluble TREM2 (sTREM2) because of the stalk region Fab′ binding site (12). It is necessary to determine the extent to which sTREM2 may influence the trapping of the radiotracer and whether binding to sTREM2 facilitates clearance or even stabilizes sTREM2 or impairs the spatial resolution of PET images. Moreover, the blood levels of radiotracer obtained through PET from segmentation represent whole blood levels, whereas the quantity of radiotracer capable of binding to a target is equivalent to the amount of unbound tracer in the plasma. Furthermore, the influence of pathology-driven changes in blood composition, particularly in conditions with strong systemic inflammation (e.g., sepsis), in which altered leukocyte dynamics and soluble TREM2 levels may impact tracer distribution, is a potential limitation.

Blood normalization represents a promising approach to reduce interindividual variability and improve quantification, particularly if the systemic availability of a radiotracer influences tissue uptake. This method may be especially useful for antibody-based or long-circulating tracers. In small animals, partial-volume effects and noise may introduce variability, whereas in humans, higher resolution could enhance robustness.

In the future, longitudinal PET imaging of TREM2 is important to determine the extent to which the radiotracer is capable of imaging alterations in myeloid cell activation throughout the course of disease progression or after antiamyloid immunotherapy.

## CONCLUSION

The analysis of TREM2 PET imaging in mice can be complicated because of radiotracer pharmacokinetics, rather high blood levels, and low signal-to-noise ratios, especially when using an antibody format as the radiotracer. Normalization of data to account for blood levels has been demonstrated to enhance the detection of TREM2 levels in brain and cardiac PET imaging. This improved methodology for TREM2 PET analysis provides a promising basis for future assessments of TREM2 PET imaging across different diseases.

## DISCLOSURE

Simon Linder and Matthias Brendel were funded by the Deutsche Forschungsgemeinschaft (DFG, German Research Foundation) with individual applications (ID 495961210, BR 4580/3-1, LI 3533/1-1) under Germany’s Excellence Strategy within the framework of the Munich Cluster for Systems Neurology (EXC 2145 SyNergy, ID 390857198 to Christian Haass and Matthias Brendel) and a Koselleck Project HA1737/16-1 (to Christian Haass). Christian Schulz and Maximilian Fischer were funded by the DZHK (German Centre for Cardiovascular Research) and the BMBF (German Ministry of Education and Research) (grants 81Z0600204 to Christian Schulz, and 81X2600256 to Maximilian Fischer). Lukas Tomas was funded by Else Kröner-Fresenius-Stiftung, the German Centre for Cardiovascular Research (DZHK), and the Munich Clinician Scientist Program (LMU). Kathryn Monroe and Dan Xia are full-time employees and shareholders of Denali Therapeutics, Inc. No other potential conflict of interest relevant to this article was reported.

## Notes

KEY POINTS **QUESTION:** Does normalizing PET images to blood levels, as opposed to the established gold standard of %ID, result in improved detection and quantification of TREM2 expression? **PERTINENT FINDINGS:** It has been demonstrated that the normalization of PET data to account for blood levels enhances the detection of TREM2 levels in the periphery of WT mice, in the brain of a mouse model of AD, and in the heart of a mouse model of myocardial infarction. **IMPLICATIONS FOR PATIENT CARE:** The use of radioligands targeting TREM2 may have the potential to enhance and complement diagnostics in preclinical and clinical studies of inflammatory drug candidates, as well as to facilitate the monitoring of therapeutic responses in antibody-based TREM2 interventions.
